# The earliest settlers' antiquity and evolutionary history of Indian populations: evidence from M2 mtDNA lineage

**DOI:** 10.1186/1471-2148-8-230

**Published:** 2008-08-11

**Authors:** Satish Kumar, PBSV Padmanabham, Rajasekhara R Ravuri, Kiran Uttaravalli, Padmaja Koneru, P Aditi Mukherjee, B Das, M Kotal, D Xaviour, SY Saheb, VR Rao

**Affiliations:** 1 Anthropological Survey of India, 27 Jawaharlal Nehru Road, Kolkata 700 016, India

## Abstract

**Background:**

The "out of Africa" model postulating single "southern route" dispersal posits arrival of "Anatomically Modern Human" to Indian subcontinent around 66–70 thousand years before present (kyBP). However the contributions and legacy of these earliest settlers in contemporary Indian populations, owing to the complex past population dynamics and later migrations has been an issue of controversy. The high frequency of mitochondrial lineage "M2" consistent with its greater age and distribution suggests that it may represent the phylogenetic signature of earliest settlers. Accordingly, we attempted to re-evaluate the impact and contribution of earliest settlers in shaping the genetic diversity and structure of contemporary Indian populations; using our newly sequenced 72 and 4 published complete mitochondrial genomes of this lineage.

**Results:**

The M2 lineage, harbouring two deep rooting subclades M2a and M2b encompasses approximately one tenth of the mtDNA pool of studied tribes. The phylogeographic spread and diversity indices of M2 and its subclades among the tribes of different geographic regions and linguistic phyla were investigated in detail. Further the reconstructed demographic history of M2 lineage as a surrogate of earliest settlers' component revealed that the demographic events with pronounced regional variations had played pivotal role in shaping the complex net of populations phylogenetic relationship in Indian subcontinent.

**Conclusion:**

Our results suggest that tribes of southern and eastern region along with Dravidian and Austro-Asiatic speakers of central India are the modern representatives of earliest settlers of subcontinent. The Last Glacial Maximum aridity and post LGM population growth mechanised some sort of homogeneity and redistribution of earliest settlers' component in India. The demic diffusion of agriculture and associated technologies around 3 kyBP, which might have marginalized hunter-gatherer, is coincidental with the decline of earliest settlers' population during this period.

## Background

The "out of Africa" model postulating a single "southern route" dispersal of "Modern human" from Horn of Africa to the Persian/Arabian Gulf and further along the tropical coast of the Indian Ocean to southeast Asia and Australasia has largely taken ground in the recent years [1-3]. This most likely involved the exodus of a founding group of several hundred individuals, who might have made the crossing from northeastern Africa, probably over the mouth of the Red Sea some time after the appearance of lineage L3 ~85,000 years ago, followed by a period of mutation and drift during which macrohaplogroups M, N, and R evolved and the ancestral L3 was lost [4]. Subsequently the same three founder macrohaplogroups, with the population expansion most likely occurring on Indian coast [5,6] shows a rapid coastal dispersal from ~66,000 years ago around the Indian Ocean littoral and on to Australasia by ~63,000 years ago [4] resulting in the non overlapping distribution of the derived haplogroups within M and N and its subclade R in south Asia, eastern Asia and Australasia.

However, the presence of the diversity of basal clades with in mtDNA macrohaplogroup M in India exceeds that in eastern Eurasia; and numerous so-called M* lineages occur in India but not in east Asia. Whereas estimated age of the M macrohaplogroup in India 54.1 thousand years (ky); [7] on the other hand is considerably low as compared to its east Eurasian counterparts (east Asia 69.3 ± 5.4 ky; Oceania 73.0 ± 7.9 ky; southeast Asia 55.7 ± 7.4 ky) [7,8]. The reason could be the molecular diversity and so as the coalescence age of the Indian M subhaplogroups themselves, which vary substantially as indicated in the studies of Sun et al. [7] and Thangaraj et al. [9].

Nested within this model, there could be two plausible scenarios:

1. The number of drift events in middle/early upper-Paleolithic populations (earliest settlers) has shaped the present day mtDNA phylogenetic structure of Indian populations. 2. Either the ancestral M existed for a minimum interval of ~30,000 to ~20,000 years, during which the younger lineages branched off sequentially or second emigrational event most likely occurring ~30,000 to ~20,000 years ago from the west of the subcontinent has given rise or brought the younger lineages, thereby accounting for the different numbers of mutations accumulated to the present.

The latter has been complicated by the fact that if not all, most of these lineages are autochthonous to India and arose essentially simultaneously from ancestral M as argued by Macaulay et al. [4]. Furthermore, since the only haplogroup of M lineages found in the substantial number to the west of the subcontinent are members of the M1 fragment, it also seems unlikely that the so appeared younger lineage of macrohaplogroup M has originated much farther west.

Owing to the aforesaid ambiguity in population structure coupled with the west Eurasian contribution into the Indian maternal gene pool as a consequence of migrations during the last 10,000 years before present (ybp) [10,11] the origin and settlement of Indian people still remains intriguing.

Of the known M lineages in India, M2 with an estimated age of ~50,000 years is the oldest [7,12] and largest sub-haplogroup, which almost accounting for one tenth of the Indian macrohaplogroup M [11,13]. The distribution of M2 is significantly more pronounced in southern part of India as compared to north, a cline similar to that of M in general [5,11]. Moreover Metspalu et al. [11] also noted that frequency of M2 among the Brahmin and Kshatriyas of Andhra Pradesh is not significantly different from that of other caste and tribal populations of the region. However it is absent among the Brahmins and Kshatriyas of the northern states of India, while the frequency reaches nearly 3 % among other caste and tribal populations of the region. The high frequency of M2 consistent with its greater age and distribution suggests that it may represent the phylogenetic signature of earliest settlers who colonized India through southern route.

To explore the past population dynamics, impact and contribution of Middle/EarlyUpper-Paleolithic settlers in shaping the genetic diversity and structure of contemporary Indian populations, we have sequenced 72 complete mitochondrial genomes of M2 lineage from 16 relic tribal populations of India.

## Results

**Figure 1:**
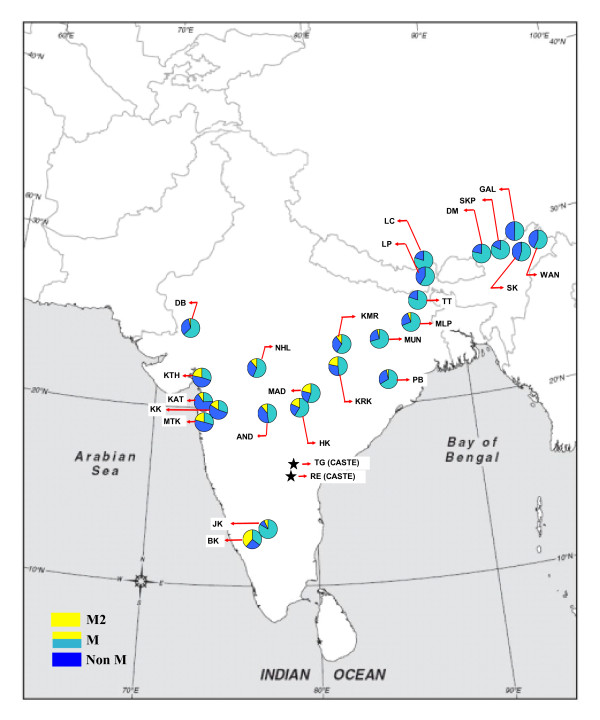
**Map of the Indian subcontinent indicating approximate locations of studied populations and mtDNA haplogroup distribution**. '*' Approximate location of the populations studied by Sun et al. [7], the mtDNA sequences of which were used in this study.

Of the screened 2768 mtDNAs from 24 tribes of India the macrohaplogroup M accounted for 69.39 %, which is consistent with the earlier reports [5,11,14]. The frequency distribution of macrohaplogroup M varies significantly (*P *<*0.0001*) among studied tribes with a cline towards southern and eastern regions of India as shown in Table 1 and Figure 1. In tribes (MaThakur, KaThakur, Kathodi, Katkari) of western region, macrohaplogroup M frequency is significantly low (~50% or less; *P *<*0.011*) as compared to the other studied regions of India. Unexpectedly Dungri Bhil representing the north-westernmost region shows a high frequency of M (76.1%) as compared to its other western counterparts.

**Table 1:** Sampling details and mtDNA lineage distribution in India.

Location	Population	Population Code	**Linguistic Affiliation**^**a**^	***n***^***b***^	Frequency (%)			
					**M**^**c**^	**Non M**^**d**^	M2	M2b
**India**				***2768***	***65.39***	***34.61***	***9.57***	***2.96***
***Western***				*629*	*47.22*	*52.78*	*13.35*	*0.00*
	DungriBhill	DB	IE	118	66.10	33.90	3.39	0.00
	Kathakur	KAT	IE	220	32.73	67.27	9.09	0.00
	Kathodi	KTH	IE	120	51.67	48.33	21.67	0.00
	Katkari	KK	IE	50	46.00	54.00	16.00	0.00
	Mathakur	MTK	IE	121	51.24	48.76	21.49	0.00
***Central***				*711*	*69.90*	*30.10*	*15.75*	*4.36*
	Andh	AND	IE	115	59.13	40.87	11.30	0.00
	Hill Kolam	HK	DR/IE	123	77.24	22.76	18.70	9.75
	Kamar	KMR	IE	111	68.47	31.53	9.91	0.00
	Korku	KRK	AA	110	70.00	30.00	21.82	15.45
	Madia	MAD	DR/IE	140	75.71	24.29	20.71	1.43
	Nihal	NHL	IE	112	66.96	33.04	10.71	0.00
***East***				*336*	*73.21*	*26.79*	*4.46*	*1.48*
	Malpaharia	MLP	AA	114	75.44	24.56	6.14	2.63
	Munda	MUN	AA/MU	102	74.51	25.49	3.92	0.98
	PaudiBhuiya	PB	DR/IE	120	70.00	30.00	3.33	0.83
***North East***				*863*	*67.32*	*32.68*	*0.12*	*0.00*
	DirangMonpa	DM	TB	100	78.00	22.00	0.00	0.00
	Gallong	GAL	TB	108	50.00	50.00	0.00	0.00
	Lachungpa	LC	TB	104	79.81	20.19	0.00	0.00
	Lepcha	LP	TB	109	58.72	41.28	0.00	0.00
	Shertukpen	SKP	TB	103	82.52	17.48	0.00	0.00
	Sonowal Kachari	SK	IE	112	56.25	43.75	0.89	0.00
	Toto	TT	TB	102	80.39	19.61	0.00	0.00
	Wanchoo	WAN	TB	125	57.60	42.40	0.00	0.00
***South***				*229*	*82.53*	*17.47*	*23.14*	*20.08*
	Betta Kuruba	BK	DR	115	74.78	25.22	39.13	35.65
	Jenu Kuruba	JK	DR	114	90.35	9.65	7.02	4.39
	Togataveera*	TG	DR	#	#	#	#	#
	Reddy*	RE	DR	#	#	#	#	#

^a ^As per Gordon 2005[47]; ^b ^Number of samples screened; ^c ^Includes all haplogroups within macrohaplogroup M; ^d ^Includes all other than macrohaplogroup M; * Caste populations data from Sun et al. [7]; # Data not available; "AA" Austro-Asiatic; "DR" Dravidian "IE" Indo-European; "MU" Mundari; "TB" Tibeto-Burman.

For the earliest settlers' component among the studied tribes, 1810 samples of macrohaplogroup M were screened for the motif that confirms haplogroup M2 within M as described in methods. Our results indicate that M2 is completely absent among the eight tribes of northeast India, expect one M2 in Sonowal Kachari. Avoiding northeast tribes, the M2 haplogroup frequency is about 13.86 % among the studied tribes. Its frequency is ~10 to ~20 % in tribes of western and central India. The frequency declines gradually to farther north and east. In southern region tribes, Betta Kuruba shows highest frequency (i.e. 39.13%) whereas the adjacent Jenu Kuruba tribe shows frequency of only 7.02 %. The distribution of subclade M2b varies greatly from complete absence among Indo-European speakers of western and central India to as high as 35.65 % among Betta Kuruba. Irrespective of region, its frequency is high (>50% of total M2) in all Dravidian speakers, except Madia tribe of central region whose linguistic affiliation is not very clear. Similarly it's frequency is high in Korku, an Austro-Asiatic tribe of central India. In eastern region M2b frequency remains low (<50% of M2).

### Defining the M2 substructure

**Figure 2:**
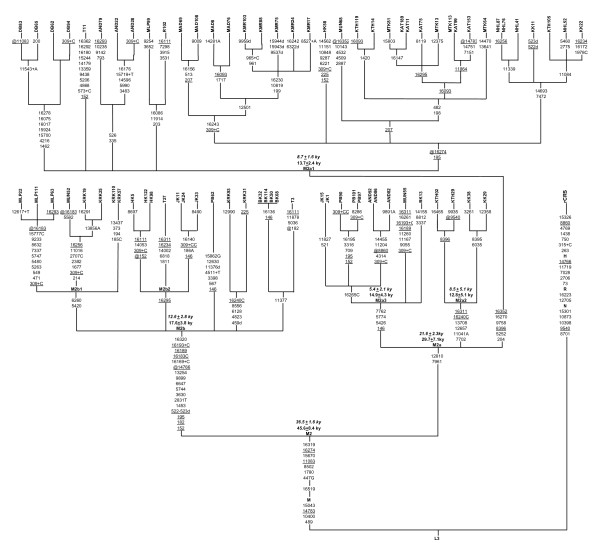
**Phylogenetic reconstruction of 76 complete mtDNAs of M2 Lineage**. Mutations were scored relative to the rCRS [58]. Sample details and population codes has been given in Table 1. Four additional complete mtDNA sequence of M2 lineage (labeled as R102, T3, T11 and T27) are acquired from published sources [7] has been used for tree reconstruction. Suffixes A, C, G, and T indicate transversions, "d" signifies a deletion and a plus sign (+) an insertion; recurrent mutations are underlined. The prefix "@" indicates back mutation. The coalescent estimates were calculated as per [16] and [17] presented as bold and Italic respectively.

The reconstructed phylogenetic tree based on our newly sequenced 72 mtDNAs of M2 haplogroup and 4 additional M2 complete sequences from the literature [7] is given in Figure 2. Out of the four defining mutations of macrohaplogroup M, one transition at nucleotide position (np) 14783 shows reversion in one of our samples. Besides the commonly occurring 16319 transition, M2 in our samples is defined by the motif 447G-1780-8502-11083-15670-16274 as also described in Kivisild et al., Rajkumar et al., Sun et al. and Thangaraj et al. [6,7,12,9]. Though one major branch in our tree lack mutation at np 16274 but due to its presence in most M2 samples of this study, as well reported elsewhere [6,7,11] we considered this mutation as a basal polymorphism of M2 as suggested in Sun et al. [7] and lack of the 16274 variant in some samples [[6,12], this study] indicates a back-mutation event. Similarly lack of mutation at np 11083 in one of our samples is also treated as reversion event.

The M2 tree shows an initial deep split into two sister clades M2a and M2b. No third clade, as indicated in Rajkumar et al. [12] has been found. The clade M2a is defined by transition at np 7961, 12810 and contains three independent basal branches M2a1, M2a2 and M2a3, in contrast to the earlier reports [6,7,11] where M2a defining motif largely constitute mutations of its sub-braches. M2a1 is defined by the motif 204-5252-8396-9758-16270-16352, in which transition at np 8396 show parallelism in two samples of M2a2 branch and transition at np 16352 shows a reversion event. M2a2 is defined by the motif of four diagnostic 7702-11041A-12657-13708 and two recurrent 16240C-16311 mutations. The branch M2a3 is defined by the motif of one recurrent np 146 and three specific 5426-5774-7762 mutations. The further divergence within these branches of M2a exhibit probable pattern of more shared haplotypes within populations of geographic proximity followed by population specific haplotypes and a few shared haplotypes among geographically apart populations.

Unlike M2a, M2b instead of early branching represented by a single deep root defined by the motif 152-182-195-522,523d-1453-2831T-3630-5744-6647-9899-13254-14766-16183C-16189-16193+C-16320 which, of late shows branching pattern similar to the sub-branches of M2a. The M2b1 defined by the transition at np 6260-5420 harbour population of eastern region. Whereas, M2b2 defined by transition at np 16295, harbours Dravidians. Other braches within M2b are more or less population specific. In this study, spread of M2b by enlarge restricted to Dravidians and tribes of eastern region. The root of M2b in our tree differs in two positions to the earlier definition of Sun et al. [7] i.e. transition at np 182 is present in all of our M2b samples so we treated this as basal mutation and lack of this in one sample of Sun et al. [7] could be better explained by reversion event, second our all M2b samples has poly 'A' at np 16180–16182 and twelve 'C's thereafter. Hence in our tree an additional 'C' at np 16184–16193 has been treated as insertion at np 16193, than transversion (A16182C) reported by Sun et al. [7].

### Age estimates and Phylogenetic implications

Coalescent age estimates were calculated by Rho (**ρ**) statistics [15] using two different mutation rates [16] and [17] shows a marginal time difference when standard deviation is taken into account, the later has been considered because of robustness in view of natural selection [17]. The average sequence divergence of the 76 M2 coding-region sequences from the root of M2 calculated as per [17] corresponds to a coalescence time estimate of 36.5 ± 1.6 thousand years (ky). The founder age estimate for Indian mtDNA lineages using M2 data, 50.0 ± 1.5 ky is well within the lower bound range of earlier estimates (i.e. sometime before 50 kyBP) of modern human dispersal into Arabia and southern Asia [1,2,4,17-21], and perhaps more close to the estimates of [17].

The two clades of M2 show differential branching patterns. M2a with coalescent age 21.6 ± 2.3 ky splits into its three deep rooting branches M2a1, M2a2 and M2a3. M2a2 is specific to Kathodi/Katkari tribe, whereas M2a1 and M2a3 encompass almost all the studied tribes. M2a1, M2a2 and M2a3 show coalescent estimates of 7 to 9 ky. The clade M2b doesn't show branching event earlier than estimated coalescence time of 12.6 ± 2.8 ky. In our samples we could not find M2b among Indo-European speakers of west and central India. The Dravidian speaking tribes of south extending up to central India and tribes of eastern region irrespective of linguistic affiliation, harbour both clades (i.e. M2a and M2b) of M2, presenting a time depth of ~37 ky.

### Diversity indices

Diversity indices and demographic parameters estimated for studied tribes are given in Table 2. The M2 lineage, haplotype diversity among Indian tribes ranged from 0.40 to 1.00 and nucleotide diversity from 0.0001 to 0.002. Though four geographical regions of India did not differ significantly (Mann-Whitney U-test) in haplotype diversity it was comparatively higher in west (0.90–1.00) followed by central (0.83–1.00), eastern (0.83–1.00) and southern tribes (0.40–1.00). Nucleotide diversity in east (0.0010–0.0019) was significantly higher than west (0.0001–0.0009; Z = 2.24, P = 0.025) and central tribes (0.00016–0.0011; Z = 2.65, P = 0.039), intermediate nucleotide diversity values were observed in south India (0.0006–0.002), they were not significantly different from west and central India (Z = 1.71; P = 0.087) or east India (Z = 0.44; P = 0.662). These patterns of genetic diversity were further strengthened by the analysis of mean pairwise differences (MPD). MPD of west (1.67–16.00) and central tribes (2.67–18.00) were significantly lower (Z = 2.41, P = 0.016) than the MPD from east (17.17–32.67), whereas MPD from south (11.20–30.00) were not significantly different from east, west and central tribes (Z = 1.39, P = 0.166). Thus observed mtDNA diversity indicate to the fact that haplotype/haplogroup frequency is a poor parameter of deep rooting ancestry rather it is the product of recent population growth. Similarly, the diversity parameters are also influenced by the past demographic events and any phylogenetic inference drawn on such parameter should keep in view the past demographic events, particularly for India where such event has been predicted previously [22].

**Table 2:** Diversity and demographic parameters deduced from complete mtDNA sequences of M2 lineage in India.

Location	Population code	***n***^***a***^	**Haplotype diversity**^**b**^	**Nucleotide diversity**^**b**^	**MPD**^**bc**^	*Fu's Fs*
**India**		***76***	***0.99 ± 0.003***	***0.0015 ± 0.0007***	***24.64 ± 10.94***	***-24.03***
***Western***		*22*	*0.99 ± 0.016*	*0.0008 ± 0.0004*	*12.96 ± 6.07*	*-7.07*
	DB	4	1.00 ± 0.177	0.0001 ± 0.0001	1.67 ± 1.21	-1.74
	KAT	5	0.90 ± 0.161	0.0002 ± 0.00016	3.80 ± 2.29	-0.13
	KTH	5	1.00 ± 0.126	0.0007 ± 0.0005	13.00 ± 7.08	0.41
	KK	4	1.00 ± 0.177	0.0009 ± 0.0006	16.00 ± 9.09	0.87
	MTK	4	1.00 ± 0.177	0.0002 ± 0.00018	4.16 ± 2.61	-0.77
***Central***		*29*	*0.99 ± 0.012*	*0.0014 ± 0.00073*	*23.97 ± 10.85*	*-4.46*
	AND	6	0.93 ± 0.122	0.0009 ± 0.0005	15.07 ± 7.87	1.49
	HK	4	0.83 ± 0.222	0.0011 ± 0.0007	18.00 ± 10.18	3.64
	KMR	5	1.00 ± 0.126	0.0002 ± 0.00016	4.00 ± 2.39	-1.72
	KRK	6	0.93 ± 0.121	0.0006 ± 0.0004	11.13 ± 5.90	1.00
	MAD	4	1.00 ± 0.177	0.0003 ± 0.0002	5.00 ± 3.06	-0.52
	NHL	4	0.83 ± 0.222	0.00016 ± 0.0001	2.67 ± 1.78	0.56
***East***		*11*	*0.98 ± 0.046*	*0.0016 ± 0.0008*	*27.38 ± 13.01*	*0.01*
	MLP	4	1.00 ± 0.177	0.0013 ± 0.0008	22.00 ± 12.37	1.22
	MUN	3	1.00 ± 0.272	0.0019 ± 0.0015	32.67 ± 19.87	2.37
	PB	4	0.83 ± 0.222	0.001 ± 0.0007	17.17 ± 9.73	3.56
***South***		*14*	*0.91 ± 0.059*	*0.0012 ± 0.0006*	*20.71 ± 9.74*	*2.77*
	BK	5	0.40 ± 0.237	0.0006 ± 0.0004	11.20 ± 6.14	7.28
	JK	5	0.80 ± 0.164	0.001 ± 0.0007	19.00 ± 10.18	5.38
	TG*	3	1.00 ± 0.272	0.002 ± 0.0014	30.00 ± 18.28	2.28
	RE*	1	#	#	#	#

^a ^Number of complete mtDNA sequences; ^b ^± Standard Deviation; ^c ^Mean Pairwise Differences; * Sequence data from Sun et al. [7]; # Unable to calculate due to single sequence.

### Past population dynamics

**Figure 3:**
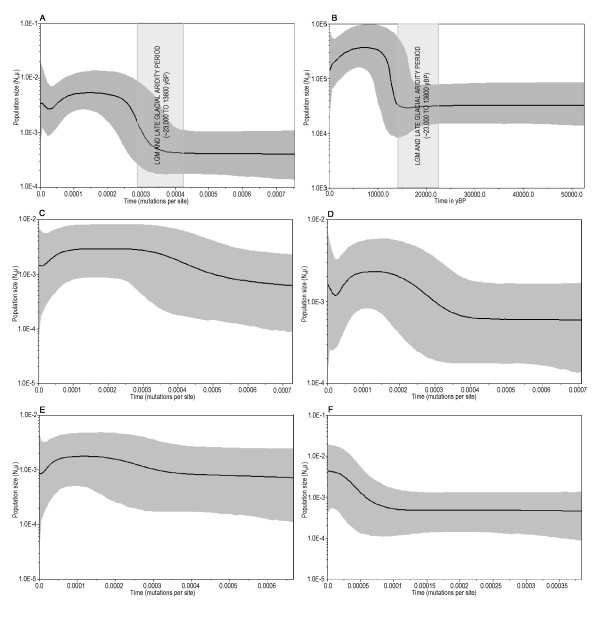
**Bayesian skyline plots showing demographic histories of earliest settlers' component**. The thick solid line is the median estimate, and the grey area overlay show the 95% highest posterior density (HPD) limits. Panel 'A'- The Bayesian skyline plot (*m *= 10) for India total, derived from complete mtDNA sequences (*n *= 76). Panel 'B'- The Bayesian skyline plot (*m *= 10) for India total, derived from coding region (577–16023) mtDNA sequences (*n *= 76). The time estimates (yBP) were calculated as per [16]. For comparison, the cold and arid period around the Last Glacial Maximum are also indicated on panel A & B. Panel 'C to F' shows Bayesian skyline plots (*m *= 10) derived from complete mtDNA sequences of eastern (n = 11), central (n = 29), southern (n = 14) and western (n = 22) regions of India respectively.

As indicated in our results and previously [22] the demographic history of populations in different geographic regions might have played pivotal role in shaping the complex net of population phylogenetic relationships in Indian subcontinent. The demographic history of M2 lineage as a surrogate of the middle/early upper Palaeolithic component of Indian populations was reconstructed using Bayesian skyline plot (BSP) [23]. Figure 3 (panel 'A') shows the BSP of M2 lineage produced using 76 complete mtDNA sequences along with plot (panel 'B') using only coding region. Although the two analyses are very similar, the second is confined to slow evolving region of mtDNA [24] which is likely to define lineages that have existed in the population prior to a putative bottleneck, thus increasing the sensitivity of BSP to detect more complex demographic trends. As the analysis is based on only single lineage it provides insight into the demographic event limiting to the age of the lineage (i.e. 37–45 kyBP). Most striking is the population decline, observed during Last Glacial Maximum i.e. 23 to 19 kyBP [25] and Late Glacial Aridity i.e. 18 to 14 kyBP [26], followed by many fold population growth in a comparatively short period of time. If such demographic event had affected the earliest settlers of India it would have resulted in several implications of phylogenetic interest. Firstly, reduction of genetic diversity across all the lineages in which, lineages with a smaller population spread would have been affected the most. Second, it might have mechanized some sort of unifying effect where smaller lineages are eliminated or at least reduced to margins of extinction and lineages of larger spread remained among all the post bottleneck populations. The Post Glacial rapid population growth achieved some sort of plateau by 7 to 3 kyBP followed by another decline which was to its maximum around ~1000 to 1500 BP. Now the question is whether the observed demographic trend was uniform throughout India or it was as complex as reported by the earlier studies [22]. A similar analysis for each studied geographical region of India is presented in panel C to F of Figure 3. Due to the small sample size in each geographic region BSP produces low resolution; however rapid post glacial population growth is evident in east, south and central India, followed by a population decline from 3 to 1 kyBP. The rapid regain after this period has been observed in central region; however such regains are marginal in other two regions. The demographic past of ancient lineage among western tribes was quite different- a population growth from ~7–8 kyBP continued to present. The negative values of Fs that differ significantly from zero indicative of population's demographic expansion [27] also support the recent population expansion in western region (Fu's Fs = -7.07; P = 0.004).

### Genetic Structure

The above results are indicative of some genetic structure in Indian populations, to investigate that, AMOVA was used (Table 3). In the total samples (model 1) 49.13% of the variance was found within populations and 50.87% among populations. Studied tribes were then grouped according to geographic proximity (model 2), linguistic affinities (model 3) and to the results suggested, namely two groups separating Indo-European speakers of west and central India from all others (model 4). Under the models 2 and 3, 45–48 % of the variance was found within populations, 36–39% among populations within groups and 13–18 % among groups. The model 4 more appropriately reflects the genetic structure with variance among groups 29.93% exceeds the variance among populations within groups 27.79%.

**Table 3:** Analysis of Molecular Variance (AMOVA).

	Model tested for population structure	Among groups		Among populations with in group		With in populations	
		Var^a^	P-value	Var^a^	P-value	Var^a^	P-value
1.	Total	-	-	50.87	<0.001	49.13	-
2.	Geographical criteria	13.66	0.043	38.81	<0.001	47.53	<0.001
3.	Linguistic criteria	17.65	0.020	36.80	<0.001	45.54	<0.001
4.	Indo-European speakers of west & central India vs. all others	29.93	<0.001	27.79	<0.001	42.28	<0.001

^a ^Variance

## Discussion

A rapid coastal migration along the "southern route" from Africa into southern Asia, some time before 50 kyBP has been strongly suggested by studies on present day world populations (especially those based on mitochondrial DNA) [1,11,18-21,28]. The founder analysis of the mtDNAs in this study suggests 50.0 ± 1.5 kyBP for such arrival which is well within the lower bound range of earlier estimates and perhaps more consistent with the earliest and most pronounced population expansion in southern Asia around 52 kyBP suggested in [28]. Magnitude of this southern Asian growth phase suggests that over half of the global human population lived in Indian subcontinent between ~45 to 20 kyBP and population size peaked at over 60% around 38kyBP [28]. These population expansion estimates are largely in agreement with high mtDNA diversity and star like non-overlapping pattern of numerous lineages of macrohaplogroup M reported previously [4,6,9,7].

Though the stage upto here is clear, the contribution and role of this sizably large earliest settlers' component in the contemporary Indian populations, coupled with later migrations during the last 10 thousand years (ky) from west and east of the subcontinent has been an issue of controversy. Cordaux et al. [22] based on the non-overlapping pattern of mtDNA phylogeny between India and east Eurasia has supported the argument of Cavalli-Sforza et al. [29] that in India the genetic traces of early migrations along the southern route were erased by the subsequent migrations, which shaped the present-day mtDNA gene pool of India. However presence of numerous autochthonous lineages in India emerging directly from the root of the founder macrohaplogroups M, N and R [4,6,7,9] during the estimated population growth period in southern Asia (~45 to 20 kyBP)[28] indicates the presence of large component of earliest settlers in the contemporary Indian populations.

In the quest of finding the carriers of the genetic legacy of the earliest settlers among the contemporary Indian populations, some previous studies on mtDNA variation by calculating nucleotide diversity and expansion time (as per methods of Slatkin et al. [30]) for different linguistic groups of India, distinguished Austro-Asiatic speaking tribes as the oldest and the carriers of the said legacy [31,32]. Basu et al. [14] also supported the view by reporting that the frequency of the ancient haplogroup M2 among the Austro-Asiatic tribal populations is as high as 19% and they lack the younger haplogroup M4. However Metspalu et al. [11], so as this study, rejects such claims as linguistic groups of India do not cluster into distinct branches of the Indian mtDNA tree, [[6,10,13], this study] calculating the beginning of expansion for those groupings is problematic, whereas lack of coding region information in Basu et al. [14] have lead to an over estimation of M2 frequency. Moreover our results indicate that M2 frequency variation among the studied tribes can be better explained by recent population expansion/demographic events than as a function of deep rooting ancestry. The nucleotide diversity though appears better parameter, is also predisposed to influence of past demographic events. The phylogenetic inferences based on such parameters should be strictly viewed in reference to the demographic events, particularly for India.

Our analysis of mtDNA variation in populations of India indicate that the Dravidian tribes extending from southern to central India and tribes of eastern India irrespective of the linguistic affiliation shows equally deep rooted M2 ancestry ~37 ky (Figure 2), comparable nucleotide diversity (Table 2) and similar past demographic history (Figure 3). However Indo-European tribes of western and central India except Kathodi/Katkari and Andh tribes harbour only M2a1 branch representing a time depth of ~8 ky. Kathodi/Katkari and Andh tribe encompasses other braches of M2a, but lacks M2b. All these Indo-European tribes shows appreciable frequency of M2 (Table 1), but they are low on nucleotide diversity (Table 2). Thus it would be highly speculative to tag any one or a group of populations based on linguistics or geography as the representatives of earliest settlers, rather it indicate to the fact that earliest settlers' component is more pronounced in the areas extending from southern to eastern India, and shows decline towards north and northwest India, a cline similar to that of M in general [[5,11], this study]. However a decline of earliest settlers' component across tribe to higher caste gradient may also be accepted in the respective regions as indicated in [13].

The time depth of M2 lineage and diversity indices in Indo-European speakers of western region extending up to central India posits the expansion of earliest settlers' component into these areas during the post Last Glacial Maximum(LGM) growth (~12 to 7 kyBP) of population (Figure 3) or perhaps little later (Figure 3-pannel 'F'). However this requires further investigation. It is only during this rapid growth; regional and population specific branching patterns appear on the more or less homogenous M2 phylogeny. The possible explanation would be the earliest settlers of India prior to this rapid population growth had lived in an extended enclave and there had been continuous gene flow across population boundaries. The second but more plausible reason of such homogeneity could be that earliest settlers by virtue of large population size during ~45 to 20 kyBP [28] and Indian ecological setting which, favoured tendency to isolate and subjugate [33] might have been differentiated into populations distributed far apart as suggested in recent studies [4,9]. But during the LGM and late glacial aridity, climate across India and south Asia generally seems to have been much more arid than present. Geomorphological indicators from the landmass of India suggest dune mobility in the northwest [34], and greatly reduced river flow in north central India during the span of time that covered the full glacial [35]. Offshore indicators of salinity (due to runoff from the land) suggest that LGM aridity was substantially greater than at present. Indicators of upwelling intensity in the Indian Ocean suggest that the summer monsoon was much weaker than present at the LGM, but reaching its weakest at around 15,800 – 12,500 C^14 ^years ago, that is 17,800-13,800 calibrated or 'real' years ago [26]. During this period of cold and more arid conditions rainforest retreated and was replaced by dry grasslands. However, some monsoon forests and woodlands in southern India and scrub, open woodland in eastern India probably existed in presently moist forest climates. This appears to be harsh conditions for an hunting gathering based subsistence, thus to fight the adverse, probably shrinking populations might have come close to each other in a more habitable area allowing a free gene flow between populations, whereas ancestral population of the Kathodi/Katkari M2a2 lineage appears to have remained isolated during this period. In the post LGM growth period, though population spread over wide geographical regions. Maternal gene flow is evident in the geographical neighbors suggesting fluidic population specific boundaries until recently at least among the tribes.

The next important event on the Indian scene is the beginnings of agriculture and use of pottery [36-41]. Cultivation of plants/agriculture diffused from the Fertile Crescent within the past 10,000 years. The steady advance beyond this stage seems however to have been primarily driven by the crop-animal complex derived from the mid-east, reaching the tip of southern India around 3 to 2 kyBP [42,43]. The diffusion of pottery traditions, which arise in response to the need to store and cook grains, shows evidence of the influences from northwest and northeast, with the western influence predominating over much of the country. Thus the Black and Red ware reflects western, while the Corded ware Chinese influence [44-46]. Two other technological innovations, known to have originated outside of India, the domestication of horse, around 6 kyBP on the shores of Black Sea in present Ukraine, and the use of iron around 5 kyBP in Anatolia in present day Turkey, appears in the Indian archeological records (around 2 kyBP) soon after the agriculture [42]. The recent study investigating the cultural or demic diffusion model of agriculture in India supported the demic diffusion model which predicts a substantial genetic input from migrating agriculturalists [47]. The advent of agriculture and perhaps migrating agriculturists brought about dramatic changes in the economy, technology and demography of human societies. Human habitat in the hunting-gathering stage was essentially on hilly, rocky and forested regions, which had ample wild plant and animal food resources. Agriculture led to the emergence of villages and towns and perhaps brought with it the division of society into occupational groups. [43]. Crop cultivation resulted in the loss of the traditional habitat of hunter-gatherers by deforestation, fragmenting and marginalizing numerous such populations, many of whom were assimilated into agriculturally based subsistence economies [48], thereby catalyzing some sort of regional similarities across tribe caste continuum. Our results on reconstructed past population demography indicating decline of earliest settlers' population (female population here) during this period in almost all the geographical regions except western (Figure 3) is consistent with the above proposition and suggests that demic diffusion of these technologies were rapid, perhaps involving large migrating populations with these technologies.

The highest frequency of east Eurasian- specific mtDNA haplogroups [11,22] and absence of M2 an earliest settlers component (Table 1) among Tibeto-Burman speaking tribes of northeastern states of India suggests that, despite the more recent migrations to India, these populations remained relatively isolated, explaining the close correlation between genetic and linguistic results [49,50]. This contrasts with the situation observed in other regions of India, where linguistic structure shows very little concordance with the genetic structures.

## Conclusion

The time depth and diversity of M2 lineage among the studied tribes suggests that the tribes of southern and eastern region along with Dravidian and Austro-Asiatic speakers of central India are the modern representatives of earliest settlers of India via proposed southern route. The LGM and late glacial (~23 to 14 kyBP), climatic conditions across India and south Asia seems to be much more arid and harsh for an hunting gathering based subsistence, thus mechanized reduction and bringing earliest settlers' population closer in a more habitable area allowing a free gene flow, followed by a rapid three fold population growth around 12-7 kyBP when climatic conditions improved, thereby inducing some sort of homogeneity and redistribution of earliest settlers' component in wide geographical regions. The next important event on the Indian scene appears to be demic diffusion of agriculture and associated technologies around 3 kyBP, resulted in the loss of the traditional habitat of hunter-gatherers by deforestation, fragmenting and marginalizing such populations, many of whom were assimilated into agricultural based subsistence economy, as evident in the decline of earliest settlers' component in all the geographical regions except western.

## Methods

### Population Samples

The approximate location of the 24 tribal populations from which 2768 mitochondrial DNAs (mtDNAs) were sampled is shown in Figure 1. Each sample comprises unrelated healthy donors from whom appropriate informed consent was obtained. The ethical clearance for the study was obtained from the organizational ethical clearance committee of Anthropological Survey of India. Further details of the whole sample collection are reported in Table 1.

### About the Populations

The population of India is culturally stratified broadly into tribal and non-tribal. It is generally accepted that the tribal people, who constitute 8.2% of the total population [51] are the original inhabitants of India [52,53]. There are an estimated 461 tribal communities in India [54], who speaks about 750 dialects [55] which can be classified into one of the following four language families: Indo-European (IE) Austro-Asiatic (AA), Dravidian (DR) and Tibeto-Burman (TB).

Considering two assumptions, (i) The M2 is one of the major matrilineal lineages contributed by the southern route migrants in the Indian populations and (ii) The tribal people being the original/earliest inhabitants of the subcontinent could have larger representation of such contribution. We have screened 24 relic tribal populations (see details in Figure 1 and Table 1) who by virtue of their habitat, socio-economic and cultural boundaries probably less influenced by the so called modern populations.

### MtDNA molecular analyses

The collected 2768 samples from 24 tribes were first screened for M macrohaplogroup. Those belongs to M (1810 in total) were typed for mtDNA motif C447G, T1780C, A8502G, G16319A which defines M2 haplogroup [6,7,9,11,12]. In our sample C447G and A8502G polymorphisms are specific to M2, whereas T1780C and G16319A are also found in the background of haplogroups other than M2 (our unpublished data).

Out of total samples screened, 265 mtDNAs belong to M2 haplogroup distributed among 17 tribes with varying frequency. Avoiding Sonowal Kachari where only one M2 sample was found, 3–6 M2 samples were randomly selected from each of the 16 tribes for complete mtDNA sequencing (72 in total).

DNA was extracted from all the collected 4–5 ml blood samples using standard phenol-chloroform methods [56] with minor modifications. For screening and complete mtDNA sequencing, DNA was PCR amplified following standard protocols and using the PCR primers and conditions of Rieder *et al*. [57]. Successful amplification was verified by electrophoresis on 1% ethidium bromide-stained agarose gels. Samples were prepared for sequencing by an ExoI/SAP cleanup to remove single-stranded DNA and unincorporated nucleotides. PCR product was sequenced with both forward and reverse primers using BigDye Terminator v3.1 sequencing kits from Applied Biosystems on an Applied Biosystems 3730 automated DNA analyzer. Contig assembly and sequence alignment was accomplished with SeqScap v2.5 software from Applied Biosystems. Mutations were scored relative to the revised Cambridge Reference Sequence (rCRS) [58] with each deviation confirmed by manual checking of electropherograms. All (n = 72) mtDNA complete genome sequences have been submitted to GenBank (accession numbers EU443443–EU443514).

### Statistical analysis

#### Phylogeny Reconstruction and Age Estimation

Besides our newly sequenced 72 mtDNAs of M2 haplogroup, 4 additional M2 complete genome sequences from the literatures [7] were employed for tree reconstruction. The phylogenetic tree was reconstructed from median-joining networks rooted to L3 using NETWORK 4.2.0.1 software [59]. The tree was checked manually to resolve homoplasies. The coalescent age estimates were calculated by Rho (**ρ**) statistics [15] and two different mutation rates i.e. one base substitution (one mutation other than indel) in the coding region (577 – 16023) per 5,140 years [16] and one synonymous transition per 6,764 year [17] calibrated on the basis of an assumed human-chimp split of 6.5 million years ago. Standard errors for coalescence estimates were calculated following Saillard et al. [15].

#### Estimates of Population Structure and evolutionary relatedness

The 76 aligned complete mtDNA sequences were analyzed for haplotype, nucleotide diversity (± SD), and mean pair-wise differences (± SD). Analyses of Molecular Variance (AMOVA) [60] were also performed to evaluate the genetic structure of the populations. The aforesaid analysis has been performed using software package ARLEQUIN version 3.0 [61].

#### Estimates of past Population Dynamics

With the prior assumption of M2 as the signature of the earliest migrants of modern humans in Indian subcontinent, we have tried to reconstruct the demographic history of earliest settlers from Most Recent Common Ancestor (MRCA), using Bayesian skyline model [23] of effective population size. Effective population size is a compound population genetic parameter generally considered linearly proportional to census population size – in this analysis, the population of breeding females. It is influenced by many factors, including local extinction, recolonization and various forms of nonrandom mating (62). The model assumes that regional populations are isolated. Estimates of effective populations were derived from the 76 complete mtDNA sequence data belonging to M2 haplogroup using Markov Chain Monte Carlo (MCMC) (63) sampling with 10 groups (m = 10) in software packages BEAST v1.4 [64] and Tracer v1.3 [65], available from http://beast.bio.ed.ac.uk/. The plots were obtained using stepwise (constant) model. The substitution model was selected by comparison of Akaike Information Criterion scores (AIC). The analysis was run for 30 million iterations with the first 10% discarded as burn-in, genealogies and model parameters were sampled at every 1,000 iterations thereafter.

## Abbreviations

AA: Austro-Asiatic; IE: Indo-European; DR: Dravidian; TB: Tibeto-Burman; ky: Thousand Years; kyBP: Thousand Years Before Present; yBP: Years Before Present; mtDNA: Mitochondrial DNA; rCRS: Revised Cambridge Reference Sequence; np: Nucleotide Position; PCR: Polymerase Chain Reaction; MPD: Mean Pairwise Differences; AMOVA: Analysis of Molecular Variance; MRCA: Most Recent Common Ancestor; MCMC: Markov Chain Monte Carlo; SD: Standard Deviation.

## Authors' contributions

SK, KU, PK and PBSVP carried out initial screening and complete mtDNA sequencing of the data. SK and RRR did sequence alignment and all the phylogenetic analysis. PAM, BD, MK, DX and SYS contributed samples. SK drafted the manuscript. VRR conceived the study, participated in its design and coordination also helped to improve the manuscript. All authors read and approved the final manuscript.
